# Pathology and Medicine

**Published:** 1858-01

**Authors:** 


### PATHOLOGY AND MEDICINE.

1. Dysenteric Ulceration and Sloughing of the Mucous Membrane of the Intestines, as a cause of Abscess of the Liver. Illustrated by twenty-two cases, occurring in the U.S. Marine Hospital, St. Louis. By *F. V. L. Brokaw, M.D.*—The difficulties of arriving at correct conclusions in the diagnosis and treatment of disease of those organs to which we cannot bring to our aid those evidences gained by a physical examination, (as auscultation and percussion in affections of the heart and lungs, the test-tube in those of the kidneys, etc.,) are lessened to a great extent when a correct history of the causes of disease to which the organs in question have been exposed can be obtained. So also those oftentimes fatal complications or secondary affections coming on in the course of disease, can only be detected by a foreknowledge of the secondary affections by which the original diseases are most liable to be followed.

Considering the liver as an immense gland, made up of a net-work of capillary vesicles investing a secretory duct, the large amount of blood which is made to pass through it in order that certain necessary changes may be produced in its quality, and bearing in mind that it is so situated in the abdominal cavity that all of the matters absorbed from the intestines, both in a state of health and disease, must of necessity pass through it before entering the general circulation, and before they can be acted upon by oxygen, it is reasonable to suppose that the absorption of noxious matter from the intestines in a state of disease, thereby producing a vitiated state of the portal blood, would induce disease of the liver.

Indeed, an association between hepatic abscess and dysentery was long since noticed, but was merely regarded as an incidental occurrence, and it was not until Dr. Budd pointed out their relation as cause and effect, that the frequency of connection between these two diseases could be accounted for; but, as Dr. Budd's explanation of the cause of the co-existence of these abscesses with dysentery, found in the *post-mortem* examinations in cases of the latter disease, has not been generally received by the profession, and having had a comparatively large number of cases of dysentery with hepatic abscess to fall under my notice during the past two years, I propose to add the results of my investigation of this subject as evidence confirmatory of the correctness of Dr. Budd's theory, and also to show more fully the great frequency of this complication in intestinal diseases, that we may be more on our guard in the treatment of bowel affections, and that we may institute such a treatment as will be most likely to secure exemption from this complication.

But previous to the consideration of dysentery as a cause of abscess of the liver, I deem it necessary to note briefly some of the other more obvious and more generally attributed causes of abscess of this organ.

Abscess of the liver may be caused by an external injury, but it is evident that this cannot occur very often, on account of the anatomical position of the liver, the organ being securely protected by the ribs.

A second attributed cause of abscess of this organ is heat, which it is argued acts as a direct stimulus to the liver, and either excites it into inflammation or strongly predisposes it to take on that condition when exposed to other causes. I have no doubt but that long-continued exposure to heat deranges the functions of the liver; the air being highly rarified, the lungs do not receive a sufficient supply of oxygen, and consequently are not able to eliminate that quantity of carbon from the blood which is requisite for the healthy action of other organs. So their functions must necessarily become deranged, and perhaps the liver suffers most, on account of its being an eliminator of carbon, and in this respect resembles, in one of its functions, the lungs. It has under these conditions an undue amount of labor to perform, which deranges its functions, and perhaps excites inflammatory action, which terminates in abscess. But I think it does so far more frequently by causing an increased flow of bile, which is often irritating in its quality; and this is most probably the cause of dysentery, which is so prevalent in hot climates; in other words, that the true origin of hepatic abscesses is to be found in dysentery, as I shall presently attempt to prove. Miasmatic influences, excesses in eating, drinking, etc., might be placed in the same category; as they act as predisposing, and sometimes as exciting, causes of dysentery.

A third cause is phlebitis. It was long since observed, that suppurative inflammation of the coats of a vein—arising from an injury to them, or from the veins becoming involved in disease, so that pus or other noxious matter could enter the venous current—was followed by the formation of abscesses in various organs—as in the lungs, liver, joints, etc.

Abscesses arising from phlebitis, which has had its origin in an injury of the coats of the veins, are most numerous, and are generally found exclusively in the lungs; and this is because those veins which are most exposed to injury—accidental, or by the knife of the surgeon—are those which return the blood immediately to the vena cava, and consequently the blood must all pass through the capillaries of the lungs before being distributed to other organs; and the pus globules or other noxious matters carried to the lungs all become arrested there, so that other organs are but little exposed to this cause.

This theory of the cause of these abscesses has been objected to, because in many cases no inflamed vein can be detected after death. But it has been observed by Arnott and Cruveilhier, "that the effects of purulent phlebitis bear no relation to the size of the inflamed vein," and that the inflamed vein may be very small, or "be within a bone."

The correctness of this theory has also been proven by the experiments of Dr. Saunders, who injected quicksilver into the veins of a dog, and observed that the animal soon had symptoms of thoracic disease which resulted in death; and, upon making an examination, he found that there were numerous small abscesses scattered through the lungs; and in the centre of each he found a globule of mercury.

Now, what is true of suppurative inflammation of those veins which return the blood to the vena cava, is also true of the veins which feed the vena portæ: as surgical operations involving these veins have been observed to be followed by abscesses of the liver.

Experiments have also been made upon these veins by Cruveilhier, who found that the injection of mercury into the mesenteric veins of a dog was followed by the formation of abscesses in the liver.

Admitting the above theory of suppurative phlebitis (the main points of which have been advanced by Dr. Budd in his work on *Diseases of the Liver*) as a cause of abscess of the liver to be true, we are prepared for the consideration of a fourth cause of hepatic abscess—dysentery.

In seventeen fatal cases of abscess of the liver, occurring in the practice of Dr. Budd, and recorded by him in his work above referred to, there was ulceration of the large intestines in ten cases.

Dr. Budd also mentions twenty-nine cases of hepatic abscesses, taken from Annesley's work on the *Diseases of India*, and in twenty-one of these there was ulceration of the intestines, and in two of the remaining eight there were marks of former ulceration.

During the past two years, while a resident physician in the Marine Hospital in this city, in twenty-seven fatal cases of dysentery in which I made *post-mortem* examinations, there were abscesses in the liver in twenty-two.

In fifteen of these cases the dysentery was of a chronic form, and the large intestines in all were more or less ulcerated and thickened. In some of the worst cases there was slight ulceration of Peyer's glands, near the termination of the ileum, and the mucous membrane of the large intestines was most extensively ulcerated throughout its entire extent, while in the less aggravated cases there were only a few small ulcers in the rectum, sigmoid flexure of the colon and cœcum.

The abscesses in these cases were fewer in number, but larger than those observed in the acute form, and had a wall of lymph built around them of more or less thickness and density.

In the acute form the mucous membrane of the large intestines was in several cases almost entirely destroyed by sloughing; the abscesses were not circumscribed by lymph, were quite numerous, and varied much in size, some being so small that they looked like mere drops of pus, while others in the same case were large enough to contain several ounces.

Ten out of the twenty-two cases had been the subjects of intermittent fever, at a longer or shorter time previous to their admission into the hospital with dysentery, while the remaining twelve had not.

I mention this because it is contended by some that these abscesses have their origin in intermittent fever, holding that the frequent recurrence of the chill induces a constant state of congestion of the liver, in consequence of which it becomes indurated, predisposing it to inflammation, and upon the occurrence of which it softens, breaks down, and results in the formation of these abscesses. Now, as there are no valves in the veins which return the blood from the chylopoietic viscera, is it not more probable that this congestion of the liver reacts upon the intestines, and in this way produces diarrhœa and dysentery? and that the abscesses found in the liver have their origin in these diseases of the bowels by their contaminating influence upon the portal blood? That the congestion of the liver induced by the cold stage of intermittent fever does react upon the intestines, and thus give rise to diarrhœa and dysentery, is, we think, established by the almost constant coexistence of intermittent fever and bowel complaints, especially when the former disease has been allowed to run on without interruption for any considerable length of time. And under such circumstances, before the bowel affection can be relieved, it is absolutely necessary first to interrupt the paroxysms of fever.

The two following cases, selected from among the ten out of twenty-two who had been the subjects of intermittent fever, will sufficiently illustrate the foregoing remarks:—

Jacob Roth, aged twenty-eight years, a stout, robust German, deck-hand on a steamboat, was admitted into the hospital September 22d, 1856, with intermittent fever, (tertian form,) the paroxysms of which were arrested by the use of quin. sulph., and he was discharged September 29th, seven days after his entrance in the hospital, and about fourteen from the time that he took the disease. He was again admitted October 24th, with acute dysentery, with which he had been suffering about twelve days. At the time of his admission his features were sunken, pale, and collapsed; had a quick, feeble pulse; frequent bloody evacuations from his bowels, attended with pain and tenesmus; pain and great tenderness upon pressure over the whole track of the large intestines, but complained of no pain in the region of the liver except when firm pressure was made upon it. He died November 8th, fourteen days after his admission in the hospital, and about twenty-six from the time that he was taken sick. In the *post-mortem* examination, the large intestines were found in a sloughing condition, the mucous membrane being entirely destroyed in large patches, leaving the muscular coat entirely bare; the lower portion of the ileum was also inflamed. The liver was a little enlarged, rather darker in color than it normally is, and scattered through its substance there were twenty-three small abscesses, varying in size from that of a pea to that of a walnut; they were not circumscribed by a deposit of lymph. The spleen was a little larger, and also darker in color than it is in its healthy state. The other viscera were healthy. In another case, in which the patient had been the subject of intermittent fever a short time previous to his admission in the hospital with dysentery, and of which he died at the end of two months, the large intestines were one ragged patch of ulceration from the ileo-cœcal valve to the extremity of the rectum. There were seventy-five small abscesses counted in the liver, varying in size from that of a mere point, containing a single drop of pus, to one that contained eight ounces.

About thirty of these abscesses could be counted upon its external surface, having the appearance of little yellow boils under the peritoneal coat; which, when cut into by the scalpel, their contents could be squeezed out, and resembled in appearance the pus of a common phlegmon. The large abscess was situated upon its upper border, and was found adherent to the diaphragm and lung, the lower lobe of which was condensed and also firmly adherent to the diaphragm. The larger abscesses in this case were circumscribed by a thin cyst of lymph, while the smaller ones had none, or at least, if they had, they were so thin that they could not be seen. That these abscesses were not consequent upon the softening of tubercles was very evident, as there were none in a crude condition, and none in the lungs or other viscera. Now, if these abscesses were consequent upon the softening of a previously indurated condition of the liver, the result of ague, why did it begin to soften at so many points? and why this coincidence with dysentery in so many cases, and how are those cases to be accounted for in which the patient had never been the subject of intermittent fever? As an illustration of those cases which had never been the subjects of intermittent fever, I will note briefly the history of two cases:—

CASE I.—William Kirkendall, aged twenty-three, a cadaveric looking individual, entered the hospital July 20th, with dysentery of three weeks' standing; features were sunken; tongue red and inclined to be dry; pulse small and frequent; tenderness upon pressure over sigmoid flexure of colon and cœcum; no pain or tenderness in the region of the liver; evacuations were fetid, mucopurulent, and attended with much tenesmus. Was treated with astringents, opiates, terebinthinate emulsions, injections, etc., but obtained no permanent relief, and died August 6th.

*Post-mortem appearances.*—Body extremely emaciated; the ravages of ulceration were most extensive, particularly in the rectum, sigmoid flexure of colon, and cœcum. (I should remark, that in the above points the destructive process of ulceration or sloughing was much aggravated in many cases, probably on account of a stasis of accumulated matter in these sac-like portions of the intestines, and by their irritating properties favoring the process of ulceration and sloughing.) The liver contained eight abscesses, averaging in size that of a walnut, and were circumscribed by a thin cyst of lymph.

CASE II.—Garret Gordon, aged twenty-nine, an Irishman, of robust habit, presented himself at the hospital August 21st, with acute dysentery, for which he had been under treatment ten days. Tongue furred, red at its tip and edges; frequent pulse; almost constant sero-sanguinolent discharges from bowels, which were very distressing, and complained of pain when pressure was made upon any part of the large intestines, also of pain in the region of the liver. Treatment same as in former case, with the addition of cut-cups and blisters to abdomen, and a free use of injections. Obtained only transitory relief, and died September 3d.

*Post-mortem.*—The mucous membrane of the large intestines had almost entirely sloughed off throughout its entire extent. The liver was somewhat congested, and its substance was thickly studded with little abscesses, from the size of a pea to that of a hazlenut; other viscera healthy.

Although in the greater number of cases of dysentery followed by hepatic abscess that have fallen under my notice the symptoms of the latter were well marked, yet in many, as has been seen in the above-reported cases, there were no symptoms by which the latter complication might be diagnosticated prior to the death of the patient. And I am convinced that in the treatment of dysentery, if a knowledge of the frequency of abscess of the liver as a secondary affection, coming on in the course of the former disease, is not had, and consequently not guarded against by treatment, that it must occur much more frequently and go on to a fatal termination without its existence ever being discovered.

In an investigation of this subject, it will be found that if we take the aforementioned seventeen cases, in which the state of the intestines were noticed by Dr. Budd, together with the twenty-nine taken from Annesley, and add them to the twenty-two cases that I have examined, we have sixty-eight cases of hepatic abscess; and in fifty-five of these there was ulceration or sloughing of the mucous membrane of the large intestines.

The great frequency with which I have noticed the connection between ulceration of the intestines and hepatic abscess, the character of the abscesses themselves, the fact that in every instance the bowel affection preceded the abscess, and that they were found exclusively in the liver, seem to justify the following conclusions:—

1st. That those abscesses of the liver found associated with dysentery—hitherto considered as a mere coincidence of these diseases—have their origin in some contamination of the portal blood, (as Dr. Budd says, work on *Diseases of the Liver*, p. 94,) "by pus formed by suppurative inflammation of one of the small intestinal veins; or by matter of other kind resulting from softening of the tissues; or by the fetid gaseous and liquid contents of the large intestines in dysentery, which must be absorbed and carried immediately to the liver."

2d. That this association, or, more properly, this complication of hepatic abscess with dysentery, is of far more frequent occurrence than has been generally supposed.

3d. That ulceration and sloughing of the mucous membrane of the large intestines in dysentery is a far more prolific cause of abscess of the liver than all others combined.—*St. Louis Medical and Surgical Journal*, November, 1857.

2. VICARIOUS SECRETION OF THE URINARY CONSTITUENTS FROM THE SKIN, FOLLOWING SECONDARY PAPULAR SYPHILIS, AND PROFUSE SPONTANEOUS PTYALISM; RECOVERY. By *Mr. Marsden*, of the Royal Free Hospital of London.—The peculiarity in the case which we record on this occasion—the notes of which were taken by Mr. W. Curran, house-surgeon to the hospital—is, that profuse ptyalism set in, no mercury having been given, and a vicarious elimination of some of the elements of the urine from the entire cutaneous surface. "Metastatic Alternation of Function" was the heading of the case as expressed by Mr. Curran, but we cannot recognize altogether such a distinction. The word "metastasis" is generally employed to express the translation of a disease from one part of the body to another; but when we desire to convey a translation of function, by which we mean the occurrence of a secretion in one part of the body instead of another, we employ the term vicarious action, or secretion—*secretio vicario*, derived from the word *vicis*, change place.

Many singular examples of this kind present themselves to our notice periodically, and, doubtless, are familiar to most physicians. The patient, in the present instance, was a young woman, aged twenty, who was admitted, with secondary syphilis, characterized by the presence of a well-marked papular eruption. After being in hospital a short time, it was noticed she had an increase in the flow of saliva, with enlargement of the sub-maxillary and sublingual glands, and swelling of the face. This condition spread to the neck and its glands, and profuse ptyalism was present. The urine had become scanty, and of low specific gravity; and it was observed that the breast, skin, and body generally possessed a well-marked urinous odor, which strongly impregnated the bed-clothes. Here, then, was the elimination of the urinary elements from the gastro-pulmonary mucous membrane and the entire integument, vicarious upon their diminution in the kidney itself. The explanation of such phenomena is, at the best of times, conjectural; and it is really difficult to give a satisfactory one. The balance in the equilibrium of secretion was restored by the use of iodide of potassium and bark, and the salivation was reduced by alum gargles. The same cause which induced the ptyalism most probably brought on the cutaneous urination. One of the actions possessed by iodide of potassium, similar in some respects to the nitrate and chlorate of potass, is that of a diuretic; in small doses, and thus acting upon the kidney, a normal state of things ensued.

We are all well aware that there is a disposition to ptyalism sometimes accompanying pregnancy. It is occasionally seen in certain diseases, such as scurvy, hysteria, some states of mania, and hydrophobia. In this last disease the patient spits incessantly. Possibly the ptyalism in the following case, by whatever cause produced, may have influenced the vicarious secretion of the urinary constituents by the skin. The following quotation from the fourth edition of Carpenter's *Physiology*, p. 622, will supply the student with information and references in relation to this curious phenomenon:—"For it seems to be established by a great mass of observations, that urine, or a fluid presenting its essential characters, may pass off by the mucous membrane of the intestinal canal, by the salivary, lachrymal, and mammary glands, by the testes, by the ears, nose, and navel, by parts of the ordinary cutaneous surface, and even by serous membranes, such as the arachnoid lining, the ventricles of the brain, the pleura, and the peritoneum. A considerable number of such cases was collected by Haller; many more were brought together by Nysten; more recently, Burdach has furnished a full summary of the most important phenomena of the kind, and Dr. Laycock has compiled a valuable collection of cases of urinary metastasis occurring as complications of hysteria."

Ann G——, aged twenty, a dull, pasty-looking girl, of heavy, sombre aspect, and strumous habit, was admitted about the latter end of April, suffering from sores on various parts of the body, partly the result of syphilis, and partly dependent on other causes: she states that she has followed the life of a street-stroller and practiced prostitution during the last three years, during which time she has frequently been obliged to sleep out a good deal at night. She used at times to drink to excess, and therein abuse herself and her health, which latter is now in a very shattered and weakly condition, and has been progressively on the decline for the last twelve months, when she first complained of any soreness or discharge. She had no bubo at the time, but noticed several small irregularities on the inner surface of the genitals, and subsequently was troubled with the itch, of which she fancies the present sores are the remains. Is not very intelligent or communicative, and seems unwilling to enter into details, but confesses that she lived indifferently, though she never wanted necessaries; and that some months ago her attention was called to a few small circular, reddish papules, which, appearing first on the face, soon assumed a mixed sandy color, which subsequently changed to dark blue, and became more prominent, crusty, and painful. On or about the 6th of June, having been previously on stomachic and tonic medicine, with porter and nutritive diet, it was observed that the flow of saliva was increased, and the neighboring glands enlarged, tender, and puffy. There was also considerable tumefaction and œdema of face, with some lividity, anorexia, and febrile heat; these were soon followed by swelling, pain, and turgescence of the neck and its glands, copious discharge of saliva, and other indications of profuse ptyalism. The urine was reported to be scanty, irritating, and altered as to color; slightly flocculent, of low specific gravity, 1·008, somewhat acid, but free from albumen; the breath, skin, mammæ, and other parts of the body smelled freely of this fluid, and the clothes and bed gave out a urinous odor. She was ordered a simple alum gargle, and a mixture of bark and iodide of potassium, and in a few days was considerably better, and is now (June 27th) quite free from uneasiness or other unpleasant symptom referrible to these parts.—*Lancet*, Nov. 21, 1857.

3. CASE OF ILEUS, WITH OPERATION, BEING AN EXTRACT FROM A PAPER ON INTESTINAL OBSTRUCTIONS. Read before the Glasgow University Medical Society, December, 1856. By *T. M'Call Anderson.*—Mr. R., aged thirty-two, had long been subject to confinement of the bowels, which he invariably relieved by a little opening medicine. However, on the 16th of May last, suffering from constipation, he took a colocynth pill and seidlitz powder, which failing to produce any effect, medical advice was called in. Various laxatives were tried, including repeated doses of croton oil, and of calomel and opium, accompanied by turpentine and assafœtida enemata, but ineffectually. The constipation at this time was the most prominent symptom. The abdomen was tympanitic, and there was slight hiccough, but he had little pain, sickness, or vomiting. This was on the 20th of May. In the evening, however, he vomited some dark-colored matter. On the 22d, enemata of tobacco were three times employed, which produced sickness and vomiting of the contents of the stomach, which smelled distinctly of the injection. Enemata of croton oil were also made use of—a mode of administering this powerful purge which I believe to be new, but which did not produce the desired effect.

On the 29th, nine ounces of fluid mercury were administered, to try its effect in forcing a passage by its weight. It may be mentioned that, at the time this was given, little confidence was placed in its efficacy by the medical men in attendance. No effect followed its use, and the pillæ carbonatis ferri saccharisatum was then had recourse to. This was given as a tonic, on the supposition that the obstruction might be owing to atony of the gut; but this was not the cause of the complaint, as the result showed it produced no effect.

Almost daily attempts were made to relieve the bowels by the long gum-elastic tube, which seemed to pass into the colon, but probably doubled on itself. On the first of June a gutta-percha tube was introduced; and on withdrawal, it was filled with a feculent-looking matter; but whether it was fæces, or matter discharged from the diseased bowel, it was impossible to say. The tube was repeatedly employed, but gave no further insight into the nature of the case, and no relief to the patient.

During the whole of this period the patient complained of no pain and no uneasiness, except what arose from the abdominal distension, which was considerable. His pulse varied from 84 to 96. Every medicine which had the least prospect of giving relief having been tried, the case was now clearly beyond the skill of the physician, and required the use of the knife. Accordingly, after consultation, an operation was agreed upon; and as the opinion was that the disease most probably lay in the sigmoid flexure of the colon, the seat of operation was on the left loin. On the 11th of June, twenty-seven days after the commencement of the obstruction, the following operation was performed by Dr. A. D. Anderson:—

The patient being put under the influence of chloroform, and being laid on his right side, at the edge of the bed, an incision was made an inch and a half above the crest of the ileum, commencing at the external margin of the erector spinæ muscle, and extending outward for about four inches. First the integument and fascia, and then layer by layer of the muscular walls were successively divided, and the descending colon was easily reached. A thick ligature was passed through it; it was drawn out and opened. At the first nothing but flatus came away; but soon after, a copious stool of half-liquid fæces, to the extent of a wash-hand basinful, was discharged. The margin of the opening in the gut was then secured to the edges of the wound, and a binder was applied round the abdomen, to support it after its distention; an aperture being left opposite the wound, which was covered with a light water-dressing. The patient was under chloroform the whole time, and said he had felt no pain; was relieved by the operation, and was comfortable. An opiate was given an hour after, and in the evening he had a good sleep, and free evacuations through the opening. Pulse 108.

On the 15th suppuration had separated the wound, but it was easy; copious stools passed without pain, and containing the crude mercury. On the 2d of July, twenty-one days after the operation, he had quite recovered from its effects, was sitting comfortably in the parlor, and all was going on well. On the 13th, however, he was suddenly seized with rigors; acute peritonitis set in, and he never rallied, but gradually sank, and died on the following morning.

On *post-mortem* examination, a malignant tumor, of a colloid character, was found at the sigmoid flexure of the colon, which proved to be the cause of the obstruction; and in the cavity of the pelvis, in close proximity to the tumor, a quantity of pus was found, which had been discharged from it, and which was the immediate cause of the fatal termination.*

Such, then, was the lamentable result of what at one time promised to be a highly successful case, and it need hardly be said that the patient's death was equally sudden and unexpected. But this need excite no surprise, since the *post-mortem* examination showed that he was laboring under a fatal complaint. The operation, although it did not save the patient's life, certainly prolonged his days, and was clearly indicated. This operation, which was proposed by Callisen, and first performed by Amussat, is an easy and safe one. There should be little hemorrhage, and the peritoneum should not be wounded, the colon being here generally uncovered by it; and it is much more likely to prove successful, and much less likely to come to a fatal termination, than the similar operation on the small intestines, where the peritoneum is unavoidably wounded, and where peritonitis is the almost inevitable result.

In conclusion, let us glance at these obstructions in a general way. Many may think with me that these cases are very rare, and not of every-day occurrence; but we have the authority of Mr. Phillips for stating that they are far more common than we can at all conceive. He has collected the results of no fewer than 2,392 *post-mortem* examinations, and he finds that in these were twenty-two cases of intestinal obstruction; or according to him—"such obstructions are observed once out of every hundred *post-mortem* inspections." Such, then, is the importance of these cases in point of frequency. Let us now look at them in point of fatality. Mr. Phillips has collected the results of one hundred and ninety-six cases, and he finds that the enormous number of one hundred and thirty-three proved fatal—that is, thirty recovered, or only one out of every five. They are, therefore, most difficult of treatment, and still more difficult to bring to a successful termination.

And lastly, as regards the treatment by operation, we find that, out of forty-four cases in which an operation was performed, fifteen were successful, or one out of every three; so that here the proportion is better than in the last case, where we included every kind of treatment, and is, therefore, in favor of operation. But while this average shows that treatment by operation is good practice, no one will be so foolhardy as to attempt anything of the kind till he has tried all the resources of the pharmacopœia, and that without avail; or till he has ascertained the probable nature of the disease, and that it is irremediable by medical treatment. Then, having satisfied himself that nothing will save the patient but an operation, the sooner he does it the better; for not only is the patient more able, in the earlier stages of the disease, to bear the shock of an operation, but the parts are in a better state; inflammation, sloughing, and other evils, have had less time to set in; and he is then much more likely to bring the case to a satisfactory conclusion.—*Glasgow Med. Journal*, Oct. 1857.

---

* The preparation is now in the Museum of the Glasgow Royal Infirmary.

